# First person – Venkatram Yellapragada

**DOI:** 10.1242/dmm.049763

**Published:** 2022-08-16

## Abstract

First Person is a series of interviews with the first authors of a selection of papers published in Disease Models & Mechanisms, helping early-career researchers promote themselves alongside their papers. Venkatram Yellapragada is first author on ‘ FGF8–FGFR1 signaling regulates human GnRH neuron differentiation in a time- and dose-dependent manner’, published in DMM. He is a doctoral candidate in the lab of Taneli Raivio at the University of Helsinki, and investigates the modeling of human diseases *in vitro* in order to identify their underlying pathophysiology.


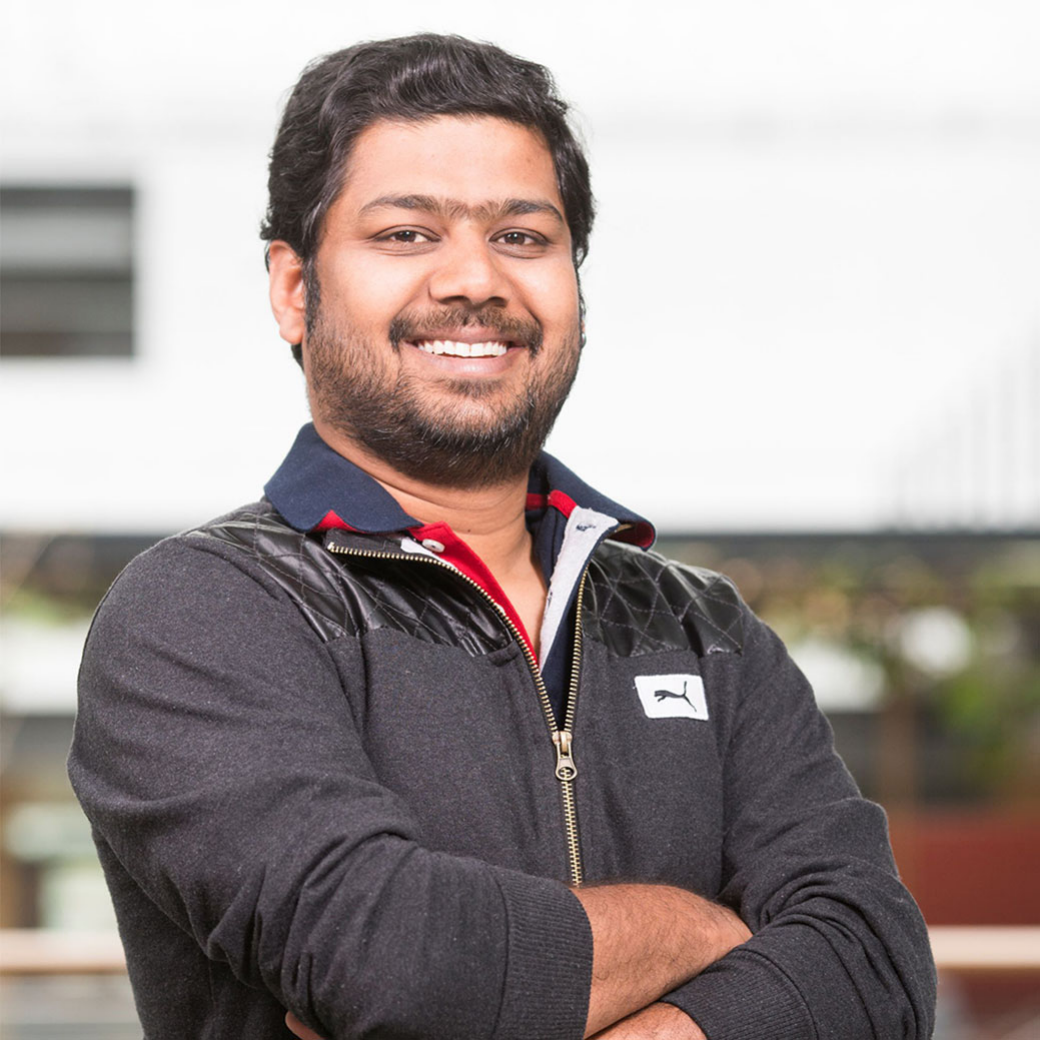


**Venkatram Yellapragada**

**How would you explain the main findings of your paper to non-scientific family and friends?**

Located in the mammalian hypothalamus, gonadotropin-releasing hormone (GnRH)-expressing neurons, i.e. GnRH neurons, are cells crucial for puberty. Development and function of GnRH neurons are complex and unique biological phenomena that require further research. Fibroblast growth factor 8 (FGF8) is an important growth factor during embryonic development and pivotal in the development of GnRH neurons. By using human pluripotent stem cells (hPSCs) as a tool, we devised a differentiation protocol to generate GnRH neurons *in vitro.* Utilizing the same, we demonstrated a dose- and time effect of FGF8 in the generation of GnRH neurons from hPSCs. We have shown that fibroblast growth factor receptor 1 (FGFR1) serves as the primary mediator for the FGF8 effect during GnRH neuron derivation. Finally, we have characterized the gene expression of FGF8-treated cells and identified dynamic changes in the expression of multiple genes during FGF8 treatment.

“The dose- and time-dependent effects of FGF8 in the generation of human GnRH neurons [...] have been demonstrated for the first time in our study.”

**What are the potential implications of these results for your field of research?**

The dose- and time-dependent effects of FGF8 in the generation of human GnRH neurons were unknown and have been demonstrated for the first time in our study. This opened the possibility of a FGF8-specification window being active during GnRH neuron development *in vivo,* which warranted further exploration. The doses and treatment times of FGF8 employed by us offer test conditions for disease modeling with several genes that are implicated in GnRH neuron development. Additionally, the gene expression profiles of FGF8-treated cells at different time-points serve as a catalogue to identify genes, pathways and networks that are activated very early during the development of GnRH neurons.

**What are the main advantages and drawbacks of the model system you have used as it relates to the disease you are investigating?**

One of the biggest advantages is the use of human pluripotent stem cells (hPSCs) since these cells are very promising human material to study development and disease. The use of human pluripotent stem cells allows us to generate GnRH neurons – which, otherwise, are difficult to access – in the lab. In fact, the hPSC-based differentiation protocol allows us to chart the developmental path of GnRH neurons *in vitro*. To mimic the precise developmental environment under which GnRH neurons emerge is a challenging task *in vitro*.

**What has surprised you the most while conducting your research?**

The origin of GnRH neurons itself is quite surprising, as these neurons develop outside the CNS and migrate all the way to the hypothalamus to exert their function. The human pluripotent stem cell-based GnRH neuron differentiation protocol we have developed employs 10 days of treatment with FGF8 and, surprisingly, we found that even 2 days of FGF8 treatment yields significant expression of gonadotropin releasing hormone 1 (*GNRH1*). It was also surprising to see that just 2 days of treatment with FGF8 changes the transcriptome of cells quite rapidly.

**Figure:**
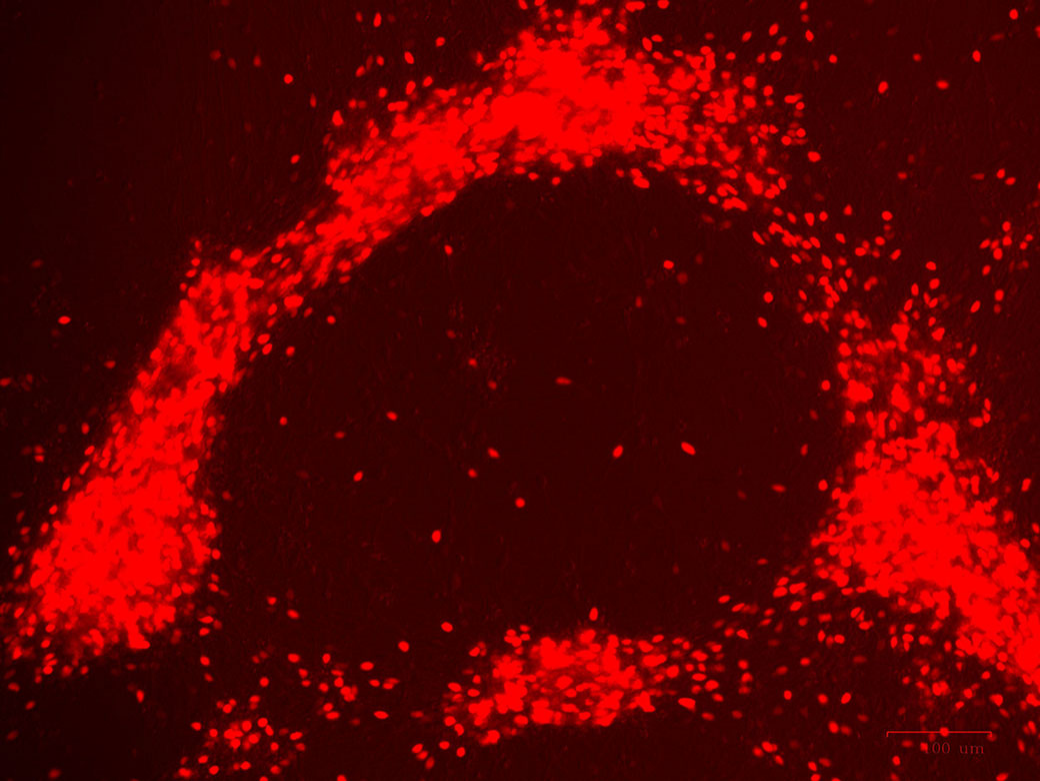
**A ring of *GNRH1*-expressing neurons.** A fluorescent live-cell image showing TdTomato-positive GnRH neurons on day 25 of the differentiation protocol, forming a ring-like structure.

**Describe what you think is the most significant challenge impacting your research at this time and how will this be addressed over the next 10 years?**

In the last decade, several technologies, such as gene editing, high-content imaging and single-cell RNA sequencing, have greatly aided the advancement of research. However, some of the remaining challenges are the availability of human material or models to study cell types that are important in understanding the complete development and functionality of GnRH neurons. To set up a precise growth and working environment for cells *in vitro* is another challenge; however, in the next decade, advancements in 3D culture methods and functional assays to characterize cells in 3D might resolve this issue to some extent.

**What changes do you think could improve the professional lives of early-career scientists?**

Availability of senior researchers within the lab/group could help early-career scientists in better planning and troubleshooting. Improving the prospects of science communication or scientific journalism imparts the value of scientific research to the outside world, which, in turn, could increase the overall professional value of scientists. Lab visits for technical specializations as part of the course curriculum is another factor to improve the professional lives of early-career scientists. Finally, one of the most important and influencing factors is an increase in research funding.

**What's next for you?**

Later this year, I will defend my PhD. I would like to follow up on my PhD research with some years of postdoctoral research to study the pathophysiology of human diseases. Eventually, I would like to transit from academia to industry or to venture into scientific journalism to communicate science.

**What do you think is something that the research communities could come up with in the future?**

Developing new databases is important for a global research community. Databases that store information regarding protocols on how to differentiate cells, the morphology of specific cell types, and optimal and efficient conditions to conduct successful gene expression (an example is single-cell RNA sequencing) are limited, and more sharing of information is necessary.
